# Limitations of Detecting Genetic Variants from the RNA Sequencing Data in Tissue and Fine-Needle Aspiration Samples

**DOI:** 10.1089/thy.2020.0307

**Published:** 2021-04-12

**Authors:** Cihan Kaya, Princesca Dorsaint, Stephanie Mercurio, Alexander M. Campbell, Kenneth Wha Eng, Marina N. Nikiforova, Olivier Elemento, Yuri E. Nikiforov, Andrea Sboner

**Affiliations:** ^1^ Division of Molecular and Genomic Pathology, University of Pittsburgh Medical Center, Pittsburgh, Pennsylvania, USA.; ^2^ Department of Physiology and Biophysics, Weill Cornell Medicine, New York, New York, USA.; ^3^ The HRH Prince Alwaleed Bin Talal Bin Abdulaziz Alsaud Institute for Computational Biomedicine, Weill Cornell Medicine, New York, New York, USA.; ^4^ Caryl and Israel Englander Institute for Precision Medicine, Weill Cornell Medicine, New York, New York, USA.; ^5^ Department of Pathology and Laboratory Medicine, Weill Cornell Medicine, New York, New York, USA.

**Keywords:** thyroid FNA, mutations, targeted NGS, RNA-Seq

## Abstract

***Background:*** Genetic profiling of resected tumor or biopsy samples is increasingly used for cancer diagnosis and therapy selection for thyroid and other cancer types. Although mutations occur in cell DNA and are typically detected using DNA sequencing, recent attempts focused on detecting pathogenic variants from RNA. The aim of this study was to determine the completeness of capturing mutations using RNA sequencing (RNA-Seq) in thyroid tissue and fine-needle aspiration (FNA) samples.

***Methods:*** To compare the detection rate of mutations between DNA sequencing and RNA-Seq, 35 tissue samples were analyzed in parallel by whole-exome DNA sequencing (WES) and whole-transcriptome RNA-Seq at two study sites. Then, DNA and RNA from 44 thyroid FNA samples and 47 tissue samples were studied using both targeted DNA sequencing and RNA-Seq.

***Results:*** Of 162 genetic variants identified by WES of DNA in 35 tissue samples, 77 (48%) were captured by RNA-Seq, with a detection rate of 49% at site 1 and 46% at site 2 and no difference between thyroid and nonthyroid samples. Targeted DNA sequencing of 91 thyroid tissue and FNA samples detected 118 pathogenic variants, of which 57 (48%) were identified by RNA-Seq. For DNA variants present at >10% allelic frequency (AF), the detection rate of RNA-Seq was 62%, and for those at low (5–10%) AF, the detection rate of RNA-Seq was 7% (*p* < 0.0001). For common oncogenes (*BRAF* and *RAS*), 94% of mutations present at >10% AF and 11% of mutations present at 5–10% AF were captured by RNA-Seq. As expected, none of *TERT* promoter mutations were identified by RNA-Seq. The rate of mutation detection by RNA-Seq was lower in FNA samples than in tissue samples (32% vs. 49%, *p* = 0.02).

***Conclusions:*** In this study, RNA-Seq analysis detected only 46–49% of pathogenic variants identifiable by sequencing of tumor DNA. Detection of mutations by RNA-Seq was more successful for mutations present at a high allelic frequency. Mutations were more often missed by RNA-Seq when present at low frequency or when tested on FNA samples. All *TERT* mutations were missed by RNA-Seq. These data suggest that RNA-Seq does not detect a significant proportion of clinically relevant mutations and should be used with caution in clinical practice for detecting DNA mutations.

## Introduction

Genetic profiling of human tumors is increasingly used to improve cancer diagnosis and prognostication and to identify potential therapeutic targets (1). For thyroid nodules, molecular testing is frequently used when fine-needle aspiration (FNA) cytology is indeterminate, and more accurate prediction of cancer probability is needed to inform patient management (2). A number of genetic alterations, such as *TERT* promoter mutations, have emerged as important prognostic markers that define the most aggressive class of thyroid cancers (3). In advanced thyroid cancer, genetic analysis is helpful to identify therapeutic targets such as *BRAF^V600E^* and *RET* mutations and *NTRK* and *ALK* gene fusions (4).

Although sequencing of DNA isolated from cells collected by FNA or tumor tissue sections is a standard approach used for detecting DNA variants, the utility of RNA converted to cDNA has been recently explored as an alternative template for sequencing and detection of DNA mutations (5–9). Such approach is based on the premise that specific DNA regions encoding genes that are expressed in the tissue of interest are transcribed into mRNA, and DNA mutations located in the coding regions of these genes can be detected by RNA sequencing (RNA-Seq).

 The use of RNA for detecting DNA variants would simplify the workflow as it would allow detection of two main classes of genetic alterations found in thyroid tumors, that is, point mutations and gene fusions, using a single RNA-based approach. However, the use of RNA for detecting gene mutations is expected to be limited to the expressed gene areas and depends on the stability and abundance of mRNA transcribed from the gene of interest in specific cell types. Indeed, several studies have demonstrated that only about half of all genetic variants detectable by sequencing of human tissue and cell line DNA could be captured using RNA-Seq (5–8). Furthermore, only limited information is available on the completeness of detection of genetic variants using the RNA-Seq data in thyroid nodules and cancer (9).

The aim of this study was to assess the completeness of capturing DNA mutations using RNA-Seq. To achieve this aim, we performed side-by-side sequencing of DNA and RNA from tissue and FNA samples and compared the detection rate of mutations in different genes and DNA regions using these two sequencing approaches.

## Materials and Methods

### Samples and study design

**FIG. 1.**
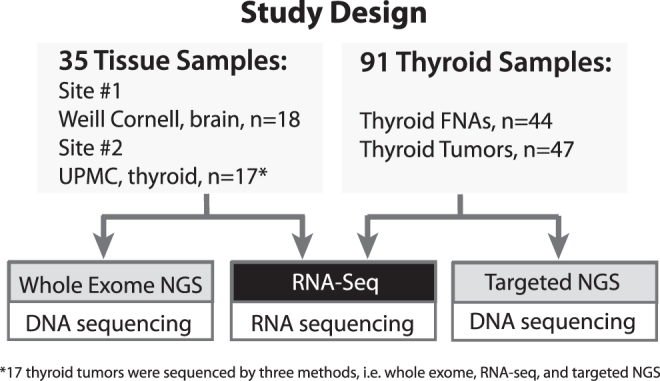
Summary of the study design.

De-identified DNA and RNA samples isolated from 35 randomly selected tissue specimens were studied using whole-exome DNA sequencing (WES) and whole-transcriptome (RNA-Seq) analysis at two study sites, the Englander Institute for Precision Medicine, Weill Cornell Medicine, New York, NY (site 1, *n* = 18, brain tumors) and the University of Pittsburgh Medical Center, Pittsburgh, PA (site 2, *n* = 17, thyroid tumors) with the approval by respective institutional review boards. In addition, DNA and RNA from 44 thyroid FNA samples and 47 thyroid tissues were studied by targeted DNA sequencing (112-gene panel) and RNA-Seq at the University of Pittsburgh Medical Center. The samples were pre-selected to represent all mutations most commonly occurring in thyroid cancer and mutations present at various levels including low level. The summary of the study design is given in Figure 1. Detected genetic alterations are summarized in Supplementary Tables S1–S3.

### Whole-exome DNA sequencing

Whole-exome DNA sequencing was performed using DNA as a template independently at the two study sites. At site 1, DNA was isolated and whole-exome DNA sequencing was performed as previously reported (10). Briefly, library preparations were performed using Agilent HaloPlex Library (Agilent Technologies, Inc., Santa Clara, CA). Sequencing was conducted on Illumina HiSeq 2500 (Illumina, Inc., San Diego, CA). Sequencing reads alignment was performed using BWA with GRC37/hg19 reference genome (11), and mutations were detected using EXaCT-1 pipeline v0.9 using patient-matched tumor/normal samples (10).

At site 2, DNA isolation and whole-exome DNA sequencing were carried out as previously described (12). Libraries were prepared using KAPA HyperPlus and xGen Exome Research Panel v1.0 (Integrated DNA Technologies, Inc., Coralville, IA). Sequencing was performed on Illumina NovaSeq using NovaSeq S1 300 Kit (Illumina) and reads alignment using BWA aligner (11) and Picard tools. For all analyses, the reference genome build GRC37/h19 was used. The aligned reads were preprocessed using Genome Analysis Tool Kit (GATK) (13). Mutations were detected using Mutect2 pipeline using “somatic only” settings. A panel of normal variants was created using data from the 1000 Genomes Project (14). The variants were annotated using ANNOVAR and filtered using quality metrics such as variant quality score recalibration, in-house database, and external databases such as Cosmic (15,16).

### Whole-transcriptome sequencing (RNA-Seq)

RNA-Seq analysis and detection of mutations were performed using protocols established at each study site. At site 1, RNA isolated from frozen specimens was prepared for RNA-Seq using TruSeq RNA Library Preparation Kit v2 or riboZero as previously described (17). Sequencing was performed on GAII, HiSeq 2000, or HiSeq 2500. RNA-Seq and data processing were conducted as previously reported (18,19). The reads were aligned using STAR_2.4.0f1 (20). SAMTOOLS v0.1.19 was used for sorting and indexing reads (21). Cufflinks (2.0.2) was used to estimate the expression values (FPKMS) (22) and GENCODE v19 GTF file for annotation (23). Since the sequenced samples from the published data sets were processed using different library preps, batch normalization was performed using ComBat (24) from sva bioconductor package (25). Mutations were identified using in-house program rnaseqmut (v0.6).

At site 2, RNA isolation and sequencing were performed as previously reported (12). To detect mutations from whole-transcriptome data, the best practice guidelines for RNA-Seq short variant discovery from the Broad Institute were used. The preprocessing was performed on raw RNA-Seq reads using STAR aligner (20) and Picard software. After recalibration of the bases on RNA-Seq reads, the variant calling was performed using HaplotypeCaller (26).

### Targeted next generation sequencing (NGS) panel

Targeted next generation sequencing analysis was performed to genotype surgically removed tissue samples (*n* = 47) and FNA samples (*n* = 44, Supplementary Table S1) from thyroid nodules using the ThyroSeq v3 GC assay, as previously described (27). The assay uses targeted amplification-based NGS technology to detect genomic alterations in 112 thyroid-related genes by sequencing DNA and RNA on the Ion GeneStudio S5 System (Thermo Fisher Scientific, Waltham, MA) according to the manufacturer's protocol. To detect mutations from targeted NGS data, the signal data from sequencing were analyzed using Torrent Suite software v5.8. For 17 thyroid tumor samples that were sequenced by whole-exome DNA sequencing, targeted DNA sequencing, and RNA-Seq, all discrepant mutations were visually inspected in addition to automated variant calling pipeline.

### Statistical analysis

Statistical analysis was performed using RStudio (1.0.136) package with R (v3.3.2) and ggplot2 (2.2.1). Comparison between the detection rate of DNA variants by different sequencing approaches was performed using *t*-test; *p*-values were two-sided and considered significant if <0.05. To evaluate the accuracy of detecting pathogenic variants by several approaches, positive percentage agreement (PPA) was calculated (28). To compare the differences in the allelic frequency (AF), root mean square (RMS) error was used. All confidence intervals were two-sided 95% and were computed using the Wald test (29).

## Results

### Detection of genetic variants by RNA-Seq compared with whole-exome DNA sequencing

To evaluate the rate of detection of cancer-related genetic variants that occur in DNA using tumor RNA, 35 tumor tissue samples were analyzed using whole-exome DNA sequencing and whole-transcriptome RNA-Seq at two participating study sites. Each study site performed detection of variants independently using their own clinical sequencing protocols and bioinformatics pipelines. Only cancer-related genetic variants were used for comparison between sequencing of DNA and RNA at each site. The results of the analysis are summarized in Table 1. Overall, the whole-exome DNA sequencing detected 162 gene variants in 35 tumor tissue samples. Of those, 77 (48%) were detected by RNA-Seq. Specifically, at site 1, 18 brain tumors were analyzed by whole-exome DNA sequencing and 32 pathogenic variants were identified, of which 15 (46%) were also detected by RNA-Seq. Site 2 analyzed 17 thyroid tumors and detected 130 variants by whole-exome DNA sequencing, of which 64 (49%) were identified by RNA-Seq. The detection rate of cancer-related genetic variants by RNA-Seq was significantly lower compared with whole-exome DNA sequencing (*p* < 0.0001). There was no difference in the detection rate of variants by RNA-Seq between the two study sites (*p* = 0.89).

**Table 1.** Detection Rates of Cancer-Related Variants by RNA Sequencing Analysis Compared with Whole-Exome DNA Sequencing

Study sites	Samples	Variants detected by WES	Variants detected by RNA-Seq	RNA-Seq (PPA*^a^*)
Site 1	18	32	15	46% (30–64%)
Site 2	17	130	64	49% (41–58%)

^a^ PPA is accuracy of detection of cancer-related genomic variants by RNA-Seq, which is calculated as a percentage of variants detected by RNA-Seq out of all genomic variants detected by DNA sequencing (WES).

PPA, positive percentage agreement; RNA-Seq, RNA sequencing; WES, whole-exome DNA sequencing.

### Detection of genetic variants by RNA-Seq compared with targeted next-generation DNA sequencing

Next, we determined the rate of detection of pathogenic genetic variants, that is, mutations, by RNA-Seq compared with targeted DNA sequencing (ThyroSeq v3 GC) in thyroid samples. Forty-seven tissue samples and 44 thyroid FNA samples were used. Among the FNA samples, 21 were diagnosed as Bethesda III, 8 as Bethesda IV, 13 as Bethesda V, and 2 as Bethesda VI. Overall, targeted DNA sequencing detected 118 gene mutations in 91 thyroid samples (Supplementary Table S2), of which 57 (48%) were identified by RNA-Seq (Table 2). Furthermore, 17 thyroid tumor samples in this group were sequenced by all three methods, that is, whole-exome DNA sequencing, targeted DNA sequencing, and RNA-Seq. All 118 mutations detected by targeted DNA sequencing were confirmed by whole-exome DNA sequencing (PPA, 100%).

**Table 2.** Detection Rates of Mutations by RNA Sequencing Analysis Compared with Targeted DNA Sequencing in 91 Thyroid Samples (47 Tissue and 44 Fine-Needle Aspiration Samples)

Gene/variants	Variants detected by targeted DNA sequencing	Variants detected by RNA-Seq	RNA-Seq (PPA*^a^*)
*BRAF*	27	13	48% (29–67%)
*RAS (NRAS, HRAS, KRAS)*	25	19	76% (59–93%)
*TERT*	23	0	0%
*TP53*	16	12	75% (54–96%)
*EIF1AX*	15	6	40%(15–65%)
*PTEN*	6	3	50% (10–90%)
*PIK3CA*	3	1	33% (0–87%)
*TSHR*	2	2	100%
*DICER1*	1	1	100%
All variants	118	57	48% (39–57%)

^a^ PPA is accuracy of detection of cancer-related genomic variants by RNA-Seq, which is calculated as a percentage of variants detected by RNA-Seq out of all genomic variants detected by DNA sequencing (targeted next generation sequencing panel).

#### Detection rate of mutations varied for different genes

We further analyzed the sequencing data to examine whether the rate of detection of mutations by RNA-Seq varies between different mutations. The most common mutations detected by targeted DNA sequencing in thyroid samples were *BRAF* (*n* = 27) and *RAS* (*n* = 25), of which RNA-Seq detected 13 (48%) of *BRAF* and 19 (75%) of *RAS* mutations (Table 2). For tumor suppressor genes *TP53* and *PTEN*, the detection rate was 75% and 50%, respectively. Other mutations were detected in expressed RNA at various rates, from 33% for *PIK3CA* and 44% for *EIF1AX* splice mutations to 100% for *TSHR* (Table 2). Finally, none of 23 samples with *TERT* promoter mutations (19 C228T, 4 C250T) were detected using RNA-Seq. If *TERT* promoter mutations were excluded, the overall detection rate of expressed DNA variants by RNA-Seq would increase from 48% to 68%.

#### Detection rate dropped for low allelic frequency mutations

Next, we determined whether the rate of detection of mutations in these samples varied for mutations present at different allelic frequencies. Allelic frequency of a mutation is calculated as a percentage of sequencing reads containing the mutation divided by total number of reads covering the locus. It allows estimating the proportion of clonal tumor cells to all (neoplastic and nonneoplastic) cells collected from each sample (by multiplying the allelic frequency by 2 as most mutations are heterozygous). This is important for clinical samples that may contain low proportion of cancer cells, such as thyroid FNA samples. This is because thyroid tumors not infrequently have significant infiltration by lymphocytes and other inflammatory cells, stromal fibroblasts, or other nonneoplastic cells that “dilute” tumor cells collected by FNA.

**FIG. 2.**
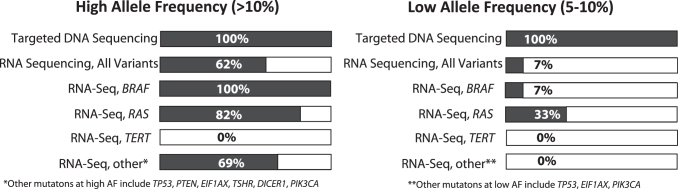
RNA-Seq detection rate of mutations that were present at high and low allelic frequencies on DNA sequencing: a study of 47 thyroid tissues and 44 thyroid FNA samples sequenced by RNA-Seq and targeted next generation sequencing panel. FNA, fine-needle aspiration; RNA-Seq, RNA sequencing.

In our group of samples, among 118 DNA mutations detected by targeted DNA sequencing, 89 had allelic frequency of >10% (i.e., a heterozygous mutation present in >20% of collected cells), which was designated as “high.” The remaining 29 variants had a “low” allelic frequency of 5–10% (i.e., were present in 10–20% of collected cells). When the rate of mutations detected by RNA-Seq was examined separately in different allelic frequency groups, we observed that 62% of high allelic frequency mutations and only 7% of low allelic frequency mutations were captured using RNA (*p* < 0.0001). These detection rates without *TERT* promoter mutation in these data sets will be 78% and 8% for “high” and “low” AF, respectively. Among the most common mutations that affect *BRAF* and *RAS* oncogenes, 94% of those present at a high (>10%) allelic frequency and 11% of those present at a low (5–10%) allelic frequency were captured by RNA-Seq. None of the *TP53*, *PIK3CA*, and *EIF1AX* mutations present at low allelic frequency were detected using the RNA-Seq. None of the *TERT* mutations were captured by RNA-Seq regardless of allelic frequency. These results are summarized in Figure 2.

#### Detection rate of mutations in thyroid FNA samples

When the detection of gene variants was examined separately in the 44 FNA samples, of 74 variants identified using targeted DNA sequencing, 27 (36%) were detected in expressed RNA (Supplementary Table S1). The detection rate would increase to 47% if the *TERT* promoter mutations are excluded. The detection of all mutations by RNA-Seq in thyroid FNA samples was significantly lower compared with tissue samples (36% vs. 49%, *p* = 0.02), and it remained significant after *TERT* mutations were excluded (47% vs. 79%, *p* = 0.008). The difference in the detection rate was due to more common presence of variants with low allelic frequency in the FNA samples (i.e., 38% of thyroid FNA samples had low-level variants compared with 7% of thyroid tumor tissues). Indeed, among mutations present in the FNA samples at an allelic frequency of >10%, the detection rate by RNA-Seq was 80% for *BRAF*, 50% for *RAS*, and 58% for other mutations. For mutations with a 5–10% allelic frequency, the detection rate by RNA-Seq dropped to 7% for *BRAF* and *RAS*, whereas *TP53* and EIF1AX mutations were not detected (Supplementary Table S1).

#### AF of mutations cannot be reliably detected by RNA-Seq

**FIG. 3.**
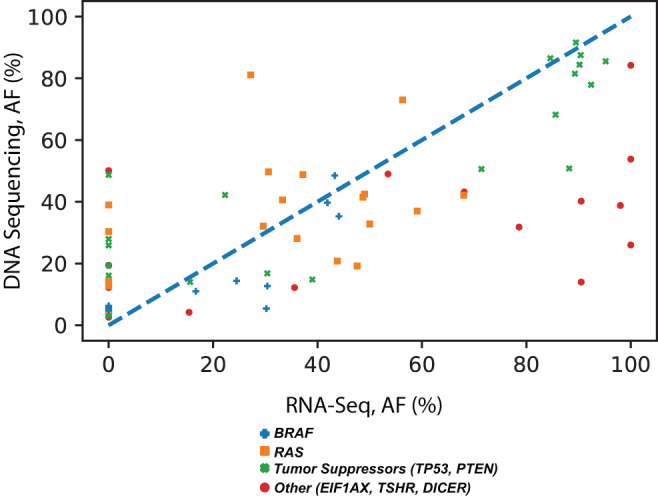
Comparison of the allelic frequencies of mutations detected by both RNA-Seq and targeted DNA sequencing in 47 thyroid tissues and 44 thyroid FNA samples. Color images are available online.

Finally, we explored the accuracy of detection of allelic frequency of gene mutations in the RNA-Seq data. Ninety-one thyroid tissue and FNA samples were used to compare the mutation allelic frequency detectable using the RNA-Seq data with those calculated using targeted DNA sequencing. Only variants that were detected by both methods were included in the comparison. Overall, correlation between the two methods was poor and showed a RMS error of 0.26 (Fig. 3). These results suggest that AFs of gene mutations may not be reliably determined using the RNA-Seq data.

## Discussion

This study assessed the completeness of detecting genetic variants using RNA-Seq and revealed that only 46–49% of pathogenic variants detectable by DNA sequencing could be captured by RNA-Seq. There was no difference in the detection rate of such variants in nonthyroid and thyroid samples and when different sequencing and detection pipelines were used at the two participating study sites.

Our findings closely recapitulate the results of previously reported studies that compared the detection of genetic variants using DNA sequencing and RNA-Seq. Indeed, in a study by Piskol *et al.*, RNA-Seq identified 40–48% of all coding variants detected by whole-genome sequencing of DNA (8). In a study by Cirulli *et al.*, 40% of exonic variants identified by whole-genome sequencing were captured using RNA-Seq (7).

There are several reasons for the limited detection of DNA variants using RNA-Seq (5–8). First, since only coding regions of genes are transcribed into mRNA, RNA-Seq cannot detect any of the variants located in noncoding DNA regions. The examples of such variants are *TERT* C228T and C250T mutations located in the promoter region of the gene, which is not transcribed. As expected, our study confirmed that all *TERT* promoter mutations were missed by RNA-Seq. *TERT* promoter mutations are important diagnostic and particularly prognostic markers for thyroid cancer (3). For example, in a nodule that contains both *RAS* and *TERT* promoter mutations, inability to detect *TERT* would yield an isolated *RAS*-positive test result, which would indicate a moderate probability of a low-risk cancer. In contrast, the correct identification of both *RAS* and *TERT* mutation would yield a high probability of high-risk cancer (30). Such limitations of RNA-Seq should be taken into account when the results of FNA analysis are intended for clinical decision-making.

The second reason for missing some genetic variants in the RNA-Seq data is that not all genes are well expressed in the source tissue, and some loss-of-function mutations may lead to decreased stability and increased degradation of respective mRNA molecules. The low number of copies of mRNA molecules makes the detection of mutations in these genes more difficult by RNA-Seq. In one study, after the analysis was restricted to those genes that were well expressed in the source tissue, the detection rate of genetic variants increased from 40% to 81% (7). In thyroid tissues, this may affect the detection of loss-of-function mutations that occur in tumor suppressor genes, such as *TP53* and *PTEN*. Among those, *TP53* mutations are of particular importance as they represent a marker of more invasive, high-risk thyroid cancers, particularly in Hürthle cell tumors (31,32).

The third reason that can affect the detection of DNA variants using RNA-Seq is related to the intrinsic complexity of the computational analysis of RNA data, which require correct mapping of RNA-Seq reads to the reference human genome (6,8). Such analysis has to account for mRNA splicing, which can be affected by different factors including mutations at the gene splice sites. One of such genes relevant to thyroid cancer is *EIF1AX*, which harbors mutations frequently affecting the splice sites between intron 5 and exon 6 of the gene (33). Indeed, in this study, only 6 of 15 (40%) *EIF1AX* mutations were captured when using RNA-Seq.

Yet another factor that may affect the detection of heterozygous mutations by RNA-Seq is related to expression balance and specific situations when the normal allelic of the gene is more highly expressed than the mutant allelic (6,34). Deviation from a 1:1 ratio of the mutant:normal allelic present in DNA in favor of the normal allelic in the sequencing reads generated by RNA-Seq can impair the detection of such mutations, particularly when they are present at a low allelic frequency. A significant drop in the detection rate of mutations present at low allelic frequency (5–10%) was observed for most mutations in this study. This confirmed previously reported findings by Angell *et al.* who observed that the detection rate of genetic variants in thyroid samples analyzed by RNA-Seq was higher for the variants present at an allelic frequency of >20% compared with those present at an allelic frequency of >5% (9). Detecting low-level mutations in thyroid FNA samples is diagnostically important, particularly for mutations that have strong association with cancer, such as *BRAF^V600E^*. This mutation, even when found at a low allelic frequency (e.g., 5%), confers a very high probability of cancer. Missing low-level *BRAF^V600E^* mutation in the FNA sample would decrease diagnostic accuracy of RNA-Seq for nodules with only partial cancer sampling or those with extensive infiltration by lymphocytes or other inflammatory cells, which would more often yield FNA samples containing a small proportion of cells carrying a given mutation.

The difference in the overall detection rate of genetic variants in thyroid samples by RNA-Seq between this study and one reported by Angell *et al.* (9) (48% vs. 74%, respectively) could be due to several reasons, including difference in mutation profiles between the tested samples. The current study included samples with a broader range of mutated genes, including *BRAF*, *RAS*, *TP53*, *PTEN*, and *TERT*, whereas in the study by Angell *et al.*, most of the samples were positive for *BRAF*, *RAS*, and *TSHR* mutations. Notably, even among thyroid samples that did not carry *TERT*, *TP53*, and other rare but clinically relevant mutations, 47/181 (26%) of mutations were missed when using RNA-Seq (9).

Similar or higher rates of genetic variants missed by RNA-Seq can be expected in thyroid FNA samples compared with the tissue samples. This is because FNA samples frequently contain a significant proportion of nonneoplastic cells, including inflammatory and stromal cells, in addition to normal thyroid cells adjacent to the tumor nodule. In the tissue samples, most of these nonneoplastic cells can be excluded before molecular analysis by selecting the most cellular tumor areas using microscopic guidance. The higher fraction of normal cells in FNA samples would lead to lowering the mutant allelic frequency, which, as discussed earlier, decreases the chance for mutations to be detected by RNA-Seq.

Finally, the findings of this study suggest that allelic frequency of mutations cannot be reliably calculated from the RNA-Seq data. This is not surprising in light of the known variability in expression levels of the normal and mutant allelics. Knowing the allelic frequency of mutations may help to provide a more specific cancer probability assessment in the tested nodules (35), although such calculations may be inaccurate based on the data generated by RNA-Seq.

In summary, the results of this study, as well as previous reports, indicate that a significant proportion of coding mutations and all noncoding *TERT* promoter mutations are missed by sequencing of RNA isolated from thyroid samples, similar to other tissue types. This should be taken into account when RNA-Seq is used in clinical practice for diagnosis, prognostication, and selection of targeted therapies for thyroid and other cancer types.

## Supplementary Material

Supplemental data

Supplemental data

Supplemental data
