# Supplementary material for: Limitations of Detecting Genetic Variants from the RNA Sequencing Data in Tissue and Fine-Needle Aspiration Samples
Source: Thyroid. 2021 Apr 12;31(4):589–95. doi: 10.1089/thy.2020.0307 (PMC8195874; doi:10.1089/thy.2020.0307)
Supplement: Supplemental data [file Supp_TableS1.docx]

**Supplemental Table 1**. Summary of mutations detected by targeted DNA sequencing and RNA-Seq in 44 thyroid FNA samples.

| Patient Sample | Gender | Age | Cytology  (Bethesda category) | Nodule Size (cm) | Variants Detected by DNA Sequencing with Allelic Fraction (%) | Variants Detected by RNA-Seq with Allelic Fraction (%) |
| --- | --- | --- | --- | --- | --- | --- |
| S-001 | M | 70 | V | 2 | *BRAF p.V600E 6.1%, NRAS p.Q61K 20.8%* | *NRAS p.Q61K 43.8%* |
| S-002 | F | 44 | V | 1.1 | *BRAF p.V600E 12.7%, TP53 p.R248W 14.8%* | *BRAF p.V600E 30.4%, TP53 p.R248W 40.8%* |
| S-003 | F | 69 | III | NA | *BRAF p.K601E 14.4%, EIF1AX p.A113_splice 12.2%,*  *TERT p.C228T 18.3%* | *BRAF p.K601E 24.5%* |
| S-004 | F | 82 | V | 1.8 | *BRAF p.V600E 6.3%, TERT C228T 19.7%* | None |
| S-005 | M | 75 | III | NA | *BRAF p.V600E 5.1%, TERT C250T 5.3%* | None |
| S-006 | F | 75 | V | 2.5 | *BRAF p.V600E 5.4%, TERT C228T 6.6%* | *BRAF p.V600E 30.2%* |
| S-007 | F | 36 | III | 1.9 | *BRAF K601E 35.3%, EIF1AX p.A113_splice 14.0%* | *BRAF K601E 44.1%* |
| S-008 | F | 76 | IV | 3.4 | *BRAF* *p.G466V 11.0%, EIF1AX p.A113_splice 40.2%* | *BRAF p.G466V 16.7%, EIF1AX p.A113_splice 90.5%* |
| S-009 | F | 36 | III | 3.4 | *BRAF p.K601E 35.3%, EIF1AX p.A113_splice 14.0%* | *BRAF p.K601E 44.1%* |
| S-010 | F | 35 | III | NA | *BRAF p.V600E 5.5%* | None |
| S-011 | F | 53 | III | 1.3 | *BRAF p.V600E 6.1%, TSHR p.M453T 4.2%* | *TSHR p.M453T 15.4%* |
| S-012 | M | 48 | VI | 2.3 | *BRAF* *p.V600E 5.4%* | None |
| S-013 | F | 63 | III | 0.6 | *BRAF* *p.V600E 4.8%* | None |
| S-014 | F | 86 | III | NA | *BRAF* *p.V600E 5.5%* | None |
| S-015 | M | 26 | III | NA | *BRAF* *p.V600E 5.1%* | None |
| S-016 | F | 33 | V | 1.7 | *BRAF* *p.V600E 5.5%* | None |
| S-017 | F | 41 | III | NA | *BRAF* *p.V600E 6.1%* | None |
| S-018 | F | 70 | V | 1.5 | *BRAF* *p.V600E 5.1%* | None |
| S-019 | M | 71 | V | 1 | *BRAF* *p.V600E 5.4%* | None |
| S-020 | F | 61 | V | 1.9 | *BRAF* *p.V600E 5.4%* | None |
| S-021 | M | 64 | V | 5 | *NRAS* *p.Q61R 32.8%, TP53* *p.P278S 50.8%, TERT* *p.C228T 35.0%* | *NRAS* *p.Q61R 50%, TP53* *p.P278S 88.2%* |
| S-022 | M | 69 | III | 1.5 | *NRAS* *p.Q61R 30.3%, EIF1AX* *p.A113_splice 53.8%, TERT* *p.C228T 41.7%* | *EIF1AX* *p.A113_splice 100%* |
| S-023 | F | 76 | IV | 2.5 | *NRAS* *p.Q61K 42.5%, EIF1AX* *p.A113_splice 38.8%, TERT* *p.C228T 49.6%* | *NRAS* *p.Q61K 52.5%, EIF1AX* *p.A113_splice 98%* |
| S-024 | F | 68 | III | 1.2 | *NRAS p.Q61K 32.1%, EIF1AX p.A113_splice 31.8%, TERT p.C228T 35.8%* | *EIF1AX p.A113_splice 78.6%* |
| S-025 | M | 60 | V | NA | *HRAS* *p.G13R 19.2%, EIF1AX p.A113_splice 26.0%, TERT* *p.C228T 15.8%* | *HRAS* *p.G13R 47.6%, EIF1AX p.A113_splice 100.0%* |
| S-026 | M | 56 | III | 2.7 | *HRAS* *p.Q61R 40.6%, EIF1AX p.A113_splice 84.2%, TERT* *p.C228T 39.4%* | *HRAS* *p.Q61R 33.3%, EIF1AX p.A113_splice 100.0%* |
| S-027 | F | 63 | III | NA | *KRAS p.G12D 12.8%, TERT* *p.C228T 13.0%* | None |
| S-028 | F | 61 | III | 2 | *HRAS p.Q61R 5.2%, TERT* *p.C228T 4.9%* | None |
| S-029 | F | 44 | III | 4 | *NRAS p.Q61R 14.1%, TERT* *p.C228T 13.8%* | None |
| S-030 | F | 54 | III | 3.7 | *HRAS p.Q61R 4.9%, TERT* *p.C228T 3.7%* | None |
| S-031 | F | 57 | IV | 3.3 | *TP53* *p.K320* 86.5%, PTEN* *p.D24Lfs*19 30.5%* | *TP53* *p.K320* 84.6%* |
| S-032 | F | 57 | IV | 3.3 | *TP53* *p.K320* 81.5%, PTEN p.R335* 84.4%* | *TP53* *p.K320* 91.6%* |
| S-033 | F | 42 | III | 1.7 | *TP53 p.R213* 25.9%, PTENp.R335* 84.4%* | *PTENp.R335* 90.2%* |
| S-034 | M | 88 | V | NA | *TP53 p.Q104* 27.9%* | None |
| S-035 | F | 53 | IV | NA | *TP53 p.R248W 16.8%* | *TP53 p.R248W 30.4%* |
| S-036 | M | 68 | IV | NA | *TP53 p.E51* 50.5%* | *TP53 p.E51* 70.4%* |
| S-037 | F | 54 | IV | 4.7 | *TP53 p.R248G 81.5%* | *TP53 p.R248G 89.3%* |
| S-038 | F | 62 | V | NA | *TP53 p.R248Q 3.1%* | None |
| S-039 | F | 68 | V | 7 | *TP53 p.R213* 91.6%* | *TP53 p.R213* 89.5%* |
| S-040 | F | 75 | III | NA | *EIF1AX p.A113_splice 19.4%, TERT* *p.C228T 5.0%* | None |
| S-041 | F | 71 | IV | 2.4 | *EIF1AX p.A113_splice 4.7%, TERT* *p.C228T 3.9%* | None |
| S-042 | M | 53 | III | 3.8 | *TERT p.C228T 47.6%* | None |
| S-043 | F | 58 | VI | NA | None | None |
| S-044 | F | 66 | III | NA | None | None |
