# Supplementary material for: Limitations of Detecting Genetic Variants from the RNA Sequencing Data in Tissue and Fine-Needle Aspiration Samples
Source: Thyroid. 2021 Apr 12;31(4):589–95. doi: 10.1089/thy.2020.0307 (PMC8195874; doi:10.1089/thy.2020.0307)
Supplement: Supplemental data [file Supp_TableS2.docx]

**Supplemental Table 2**. Summary of mutations detected by targeted DNA sequencing in 91 thyroid samples (47 tissue and 44 FNA).

| **Gene** | **cDNA change** | **Amino Acid change** | **Chromosome** | **Position** | **Transcript** | **Number of samples** |
| --- | --- | --- | --- | --- | --- | --- |
| *BRAF* | 1799T>A | V600E | 7 | 140453136 | NM_004333.4 | 23 |
| *BRAF* | 1801A>G | K601E | 7 | 140453134 | NM_004333.4 | 3 |
| *BRAF* | 1397G>T | G466V | 7 | 140481411 | NM_004333.4 | 1 |
| *NRAS* | 182A>G | Q61R | 1 | 115256529 | NM_002524.4 | 13 |
| *NRAS* | 181C>A | Q61K | 1 | 115256530 | NM_002524.4 | 4 |
| *HRAS* | 182A>G | Q61R | 11 | 533874 | NM_005343.2 | 4 |
| *HRAS* | 181C>A | Q61K | 11 | 533875 | NM_005343.2 | 2 |
| *HRAS* | 37G>C | G13R | 11 | 534286 | NM_005343.2 | 1 |
| *KRAS* | 35G>A | G12D | 12 | 25398284 | NM_004985.3 | 1 |
| *EIF1AX* | 338-2A>T | A113_splice | X | 20148727 | NM_001412.3 | 15 |
| *TERT* | 1-124C>T | C228T | 5 | 1295228 | NM_198253.2 | 19 |
| *TERT* | 1-146C>T | C250T | 5 | 1295250 | NM_198253.2 | 4 |
| *TP53* | 821T>C | V274A | 17 | 7577117 | NM_000546.5 | 1 |
| *TP53* | 454C>T | P152S | 17 | 7578476 | NM_000546.5 | 1 |
| *TP53* | 524G>A | R175H | 17 | 7578406 | NM_000546.5 | 1 |
| *TP53* | 818G>A | R273H | 17 | 7577120 | NM_000546.5 | 1 |
| *TP53* | 596G>A | G199E | 17 | 7578253 | NM_000546.5 | 1 |
| *TP53* | 958A>T | K320* | 17 | 7576888 | NM_000546.5 | 2 |
| *TP53* | 832C>T | P278S | 17 | 7577106 | NM_000546.5 | 1 |
| *TP53* | 790C>G | L264V | 17 | 7577148 | NM_000546.5 | 1 |
| *TP53* | 742C>T | R248W | 17 | 7577539 | NM_000546.5 | 2 |
| *TP53* | 151G>T | E51* | 17 | 7579536 | NM_000546.5 | 1 |
| *TP53* | 637C>T | R213* | 17 | 7578212 | NM_000546.5 | 2 |
| *TP53* | 310C>T | Q104* | 17 | 7579377 | NM_000546.5 | 1 |
| *TP53* | 742C>G | R248G | 17 | 7577539 | NM_000546.5 | 1 |
| *PTEN* | 955dup | T319Nfs*6 | 10 | 89720803 | NM_000314.6 | 1 |
| *PTEN* | 70_71del | D24Lfs*19 | 10 | 89624295 | NM_000314.6 | 2 |
| *PTEN* | 1003C>T | R335* | 10 | 89720852 | NM_000314.6 | 1 |
| *PTEN* | 494G>A | G165E | 10 | 89711876 | NM_000314.6 | 1 |
| *PTEN* | 202T>C | Y68H | 10 | 89685307 | NM_000314.6 | 1 |
| *PIK3CA* | 1090G>A | G364R | 3 | 178922321 | NM_006218.2 | 2 |
| *PIK3CA* | 3140A>G | H1047R | 3 | 178952085 | NM_006218.2 | 1 |
| *TSHR* | 1887G>T | L629F | 14 | 81610289 | NM_000369.2 | 1 |
| *TSHR* | 1358T>C | M453T | 14 | 81609760 | NM_000369.2 | 1 |
| *DICER1* | 5439G>C | E1813D | 14 | 95557628 | NM_030621.4 | 1 |
