# Supplementary material for: Limitations of Detecting Genetic Variants from the RNA Sequencing Data in Tissue and Fine-Needle Aspiration Samples
Source: Thyroid. 2021 Apr 12;31(4):589–95. doi: 10.1089/thy.2020.0307 (PMC8195874; doi:10.1089/thy.2020.0307)
Supplement: Supplemental data [file Supp_TableS3.docx]

**Supplemental Table 3**. Summary of mutations detected by whole exome sequencing in 18 brain tissue samples.

| **Gene** | **Amino Acid change** | **Number of samples** |
| --- | --- | --- |
| *BCL11A* | P367S | 1 |
| *BRAF* | V600E | 1 |
| *BRAF* | V600L | 1 |
| *CASP9* | P164Q | 1 |
| *CDKN2A* | G101E | 1 |
| *CDKN2A* | G101V | 1 |
| *CNKSR1* | S530L | 1 |
| *EGFR* | L62R | 1 |
| *FGFR3* | D160A | 2 |
| *KIAA0195* | Q376* | 1 |
| *KMT2D* | R3321* | 1 |
| *KMT2D* | Q4364R | 1 |
| *NIN* | E1168A | 1 |
| *PDE4DIP* | R25H | 1 |
| *PHLDB3* | R4Q | 1 |
| *PLAG1* | T378I | 1 |
| *PTEN* | C105F | 2 |
| *SPOP* | F125V | 1 |
| *TP53* | Y236C | 2 |
| *TP53* | E286V | 1 |
| *TP53* | E286G | 1 |
| *TP53* | E286K | 1 |
| *TP53* | I195T | 2 |
| *TP53* | R273H | 1 |
| *TP53* | R273C | 1 |
| *TTC14* | H690Y | 1 |
| *UNC5D* | R554C | 1 |
| *ZP3* | R345T | 1 |
